# Poor Clinical Sensitivity of Rapid Antigen Test for Influenza A Pandemic (H1N1) 2009 Virus

**DOI:** 10.3201/eid1510.091186

**Published:** 2009-10

**Authors:** Jan Felix Drexler, Angelika Helmer, Heike Kirberg, Ulrike Reber, Marcus Panning, Marcel Müller, Katja Höfling, Bertfried Matz, Christian Drosten, Anna Maria Eis-Hübinger

**Affiliations:** Institute of Virology, Bonn, Germany

**Keywords:** Influenza, viruses, influenza virus A (H1N1), rapid antigen-based test, real-time PCR, sensitivity, communicable diseases, Germany, influenza A pandemic (H1N1) 2009 virus, dispatch

## Abstract

Influenza A pandemic (H1N1) 2009 virus RNA was detected by reverse transcription–PCR in 144 clinical samples from Bonn, Germany. A common rapid antigen–based test detected the virus in only 11.1% of these samples. The paramount feature of rapid test–positive samples was high virus concentration. Antigen-based rapid tests appear unsuitable for virologic diagnostics in the current pandemic.

In April 2009, a novel human influenza virus A (H1N1) variant, influenza A pandemic (H1N1) 2009 virus, was identified in Mexico and the United States (*1*). Efficient human-to-human transmission facilitated global spread of this virus. On June 11, 2009, the World Health Organization (WHO) raised its pandemic alert level to Phase 6, indicating ongoing pandemic transmission. By July 27, WHO had registered 134,503 laboratory-confirmed cases and 816 confirmed deaths caused by pandemic (H1N1) 2009 virus infection worldwide (*2*).

In Germany, 5,324 cases were confirmed by July 30 (*3*). Almost 50% (n = 2,184) of these cases occurred in the federal state of North Rhine-Westphalia in western Germany, where our institution is located. As of July 30, we had tested 1,838 suspected cases and confirmed 221. All testing was based on real-time reverse transcription–PCR (RT-PCR) specific for the hemagglutinin (HA) gene of pandemic (H1N1) 2009 virus in clinical specimens. Although the real-time RT-PCR format provides considerably decreased turnaround times in molecular diagnostics, delays associated with shipping of samples and laboratory-based testing are a concern when many patients have to be seen in short time. Antigen-based rapid assays can be used as bedside tests and have been successfully applied in studies of influenza caused by the seasonal strains A (H1N1) and A (H3N2) (*4*).

## The Study

To evaluate the clinical applicability of a widely distributed rapid test in patients with pandemic (H1N1) 2009 virus, we retrospectively tested 144 PCR-positive clinical specimens from 144 different patients with the BinaxNOW Influenza A&B Rapid Test (Inverness Medical, Cologne, Germany). The assay uses monoclonal antibodies directed against the influenza A and B nucleoproteins (NP).

During preanalytical preparation, all of our samples (nasal and throat swabs) had been eluted from the swab in 500 µL of 0.9% sodium chloride solution and used for RNA extraction. The remaining part of the suspension was immediately stored at −70°C until use in this study. Before inclusion in the study, presence of pandemic (H1N1) 2009 virus in RNA extracts of all 144 samples was confirmed with a second pandemic (H1N1) 2009 virus–specific real-time RT-PCR targeting the matrix gene. Virus concentrations in samples were determined from the RNA extract without thawing the original sample, using real-time RT-PCR for the HA gene. Absolute quantification was done using photometrically quantified RNA in vitro transcripts, according to methodology described earlier (*5*).

The BinaxNOW assay was used exactly according to the manufacturer’s instructions on 100 µL of freshly thawed original sample. Of 144 PCR-confirmed cases, only 16 could be detected by using the rapid antigen–based test, corresponding to a sensitivity of merely 11.1% (95% confidence interval 6.7–17.7). Samples that yielded positive results in the rapid test had a median concentration of 4,570,880 RNA copies/mL of suspension (range 5,370–74,131,020) (Figure). This contrasted with 20,089 (range 120–64,565,420) median viral RNA copies in the rapid test–negative group. This difference was highly significant at p<0.001 (1-way analysis of variance [ANOVA], F = 38.824, done on logarithmic RNA concentrations).

**Figure:**
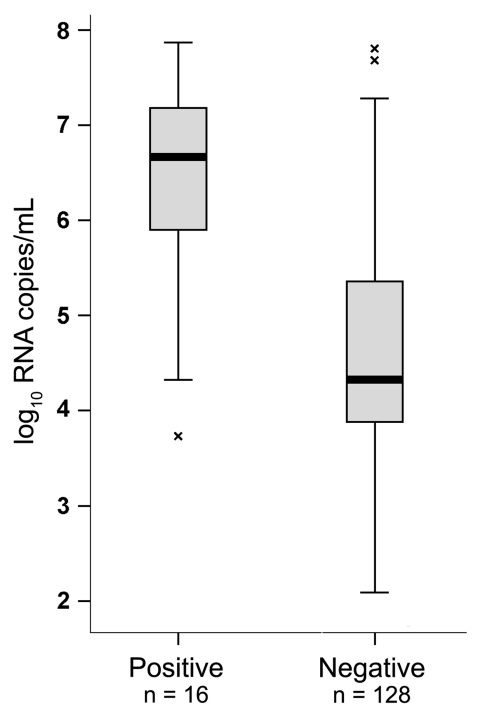
Influenza A pandemic (H1N1) 2009 virus variant RNA concentrations in rapid test–positive and –negative patients, Germany, 2009. Viral RNA concentration is compared between patients yielding positive and negative results in the BinaxNOW (Inverness Medical, Cologne, Germany) antigen-based rapid test. Boxplots were produced using SPSS, version 13.0 (SPSS, Chicago, IL, USA). The box shows the median and interquartile range (box length). The whiskers represent an extension of the 25th or 75th percentiles by 1.5 × interquartile range. Data points beyond the whisker range are considered as outliers and marked as crosses.

The BinaxNOW rapid antigen–based assay was used in our institution during the preceding 2 influenza seasons (2007–08 and 2008–09). The 2007–08 epidemic was driven almost exclusively by seasonal influenza virus A (H1N1) in Germany, whereas the 2008–09 epidemic was mostly caused by influenza virus A (H3N2) (*6*). The sensitivity of BinaxNOW in comparison with a standard RT-PCR assay (*7*) was 37.5% (6 rapid test positives in 16 PCR positives) and 51.9% (14 rapid test positives in 27 PCR positives) in the influenza seasons 2007–08 and 2008–09, respectively. These results agree with published data on the application of BinaxNow and other rapid antigen–based tests (*8*–*10*). Similar to pandemic (H1N1) 2009 virus, virus concentration was the main determinant for a positive rapid test during both earlier influenza seasons, although slightly less marked than in the current pandemic (2007–08, median RNA copies/mL 74,131,020 [range 3,981,070–8,709,635,900] and 346,740 [range 2,450–5,495,410] in rapid test–positive vs. –negative patients, respectively [1-way ANOVA, p<0.001, F = 21.127]; 2008–09, median RNA copies 19,498,446 [range 138,040–1,737,800,830] and 120,230 [range 38,900–100,000,000] in rapid test–positive vs. –negative patients, respectively [1-way ANOVA, p = 0.001, F = 15.659]).

## Conclusions

Because children tend to have higher influenza virus shedding than adults, the overall better sensitivity of the antigen-based rapid test in preceding influenza A seasons might be due to sample selection effects because the current pandemic leads to higher percentages of adult samples than does regular seasons. The median age in our cohort was 18 years (range 1–59 years), with no significant age difference between rapid test–positive and –negative patients (1-way ANOVA, p = 0.246, F = 1.356). Most of the samples from earlier influenza A seasons originated from children <7 years of age (2008, median 3 years, range 1–17 years; 2009, median 4 years, range 1–67 years).

In agreement with our study, a recent report by Faux at al. on the detection of pandemic (H1N1) 2009 virus by rapid antigen test found low clinical sensitivity for the QuickVue Influenza assay (Quidel, San Diego, CA, USA) in comparison to RT-PCR (*11*). These authors found clinical sensitivity of QuickVue to be ≈51%. Notably, earlier studies on seasonal influenza have identified 27% clinical sensitivity for the same assay in comparison to RT-PCR (*12*), and the higher sensitivity for pandemic (H1N1) 2009 virus observed by Faux et al. might be due either to a rather small sample size in this early study (39 patients) or a different composition of the cohort (military personnel) as opposed to our study. Nevertheless, our clinical observations are supported by recent reports suggesting low analytical sensitivity of antigen-based assays on cultured pandemic (H1N1) 2009 virus (*13*,*14*) and clinical material analyzed using 2 different antigen-based rapid tests, virus culture, and a Luminex-based multiplex assay (*15*). A synopsis of data suggests clearly that testing of patients suspected of pandemic (H1N1) 2009 infection with antigen-based assays may produce misleading results in clinical practice. Application of such assays should be discouraged in favor of continued molecular diagnostics.
